# Examining family and facility characteristics associated with family satisfaction in assisted living: the Minnesota Assisted Living Report Card project

**DOI:** 10.1093/geroni/igag067

**Published:** 2026-06-23

**Authors:** Tetyana P Shippee, Romil R Parikh, Molin Ji, Timothy J Beebe, Mark Woodhouse, Martina Johnson, Julie Angert, Lauren Glass

**Affiliations:** Division of Health Policy and Management, University of Minnesota School of Public Health, Minneapolis, Minnesota, United States; Division of Health Policy and Management, University of Minnesota School of Public Health, Minneapolis, Minnesota, United States; Division of Health Policy and Management, University of Minnesota School of Public Health, Minneapolis, Minnesota, United States; Division of Health Policy and Management, University of Minnesota School of Public Health, Minneapolis, Minnesota, United States; Division of Health Policy and Management, University of Minnesota School of Public Health, Minneapolis, Minnesota, United States; Aging and Adult Services Division, Minnesota Department of Human Services, St. Paul, Minnesota, United States; Aging and Adult Services Division, Minnesota Department of Human Services, St. Paul, Minnesota, United States; Aging and Adult Services Division, Minnesota Department of Human Services, St. Paul, Minnesota, United States

**Keywords:** Quality measurement, Person-reported measures, Person-centered care, Long-term care

## Abstract

**Background and Objectives:**

Family members play an essential role in monitoring and advocating for quality of care in assisted living (AL) communities. Despite their importance, few population-based studies have examined how family and facility characteristics influence family satisfaction in AL. We evaluated family and facility characteristics associated with family satisfaction in AL.

**Methods:**

Using cross-sectional survey data from the Minnesota Assisted Living Report Card project (2024), we identified resident- and facility-level predictors of AL resident-reported quality of life in 8 domains, including experience, choice, needs, housekeeping, food, environment, staff, and overall satisfaction, using multivariable linear mixed regression with facility-level random intercept to account for facility-level clustering.

**Results:**

Among 15 320 family member respondents in licensed AL facilities with more than 5 beds (51.1% of age <65 years, 65.4% female, 92.3% White), mean domain scores ranged from 67.2 (Food) to 82.1 (Overall), indicating generally positive perceptions. Across all domains, lower satisfaction was observed among respondents who are younger, people of color, and female, and among spouses or siblings of residents. Families visiting or communicating less frequently tended to report lower satisfaction with choice and overall satisfaction. Smaller and non-profit facilities received higher ratings across most domains, while facilities in the Twin Cities metropolitan area scored higher than those in the rest of the state.

**Discussion and Implications:**

Family satisfaction in AL reflects both family composition and facility context. Policymakers and providers can leverage family satisfaction metrics to inform targeted quality improvement and promote equitable, family-centered care across the AL sector.

Innovation and Translational Significance: This study leverages one of the only statewide Assisted Living Report Card systems in the United States to link family-reported satisfaction with facility-level characteristics at scale. By integrating multilevel survey data across nearly 1000 facilities, we identify actionable family and organizational factors associated with satisfaction, highlighting disparities by age, race, and facility context. Findings translate directly to quality improvement, public reporting, and policy strategies aimed at advancing equitable, family-centered care in AL.

## Introduction

Assisted living (AL) communities are a rapidly growing segment of long-term services and supports in the United States, serving more than 800 000 older adults nationwide.^1^ AL offers a residential care model emphasizing autonomy, social engagement, and a homelike environment while providing supportive services for individuals who do not require the intensity of nursing home care.^2^^,^^3^ As the population ages and preferences shift toward less institutional settings, ensuring high-quality care and experiences in AL has become a central priority for providers, policymakers, and families alike.^2^^,^^3^

Family members play an essential role in person-centered care planning and resident advocacy within AL settings. They often supplement care provision, coordinate with staff, and monitor quality on behalf of residents who may have cognitive or functional limitations.^4–6^ Consequently, family satisfaction is an important indicator of overall care quality, capturing dimensions of interpersonal communication, responsiveness, environment, and trust that may not be fully reflected in traditional quality metrics.^3–6^ Despite its importance, few studies have systematically examined factors influencing family satisfaction in AL, particularly using population-based data that link family perspectives with resident and facility characteristics.^3^ Understanding the multilevel determinants of family satisfaction in AL can guide targeted quality improvement efforts and inform state and federal policy initiatives aimed at promoting resident- and family-centered care.

Existing research has identified several domains that may influence family satisfaction in residential care, including facility size, staffing adequacy, organizational culture, and communication practices.^3–5^^,^^7^^,^^8^ However, much of the evidence comes from small, regional studies or national surveys that lack facility-level linkage, limiting insight into how family and facility factors jointly shape satisfaction.^3–5^^,^^7^^,^^8^ Moreover, most prior work has focused on nursing homes rather than AL, where the regulatory environment, resident mix, and service models differ substantially.^6–8^ Minnesota provides a unique context for studying AL quality, given its robust statewide Assisted Living Report Card initiative that collects data from residents, families, and facilities.^9^ This system enables linkage of individual family responses to facility characteristics, offering a rare opportunity to examine how family perceptions vary across diverse AL settings.^10^ In this study, we used data from the Minnesota Assisted Living Report Card to identify family and facility characteristics associated with family satisfaction across multiple quality domains. By integrating individual- and facility-level data, we aimed to provide a comprehensive, multilevel understanding of the factors that influence family satisfaction in AL. Our findings can inform targeted strategies for improving communication, care practices, and quality oversight in this vital sector of long-term care.

## Methods

### Data source

The Minnesota Department of Human Services and the Minnesota Board on Aging maintain a public Assisted Living Report Card website that reports on quality across AL settings statewide.^9^ A key component of this report card is the AL Family Satisfaction Survey (FSS).^9^^,^^10^ We used data from the 2024 FSS, which was administered to family members of residents in licensed AL facilities throughout Minnesota. Facilities were eligible to participate if they held an AL license and had the capacity to serve 5 or more residents. Of the 1198 eligible facilities, representatives from 982 facilities completed the survey (82% facility response rate). Standardized surveys were administered through 3 administration methods (mailed survey, online completion, and phone administration). The goal was to obtain enough completed surveys per facility to achieve a representative sample based on facility size and a ±6% margin of error. If a facility did not have the required number of completed surveys online or via mail, follow-up phone interviews were conducted to meet the margin of error target established a priori. The average number of family surveys completed at each facility was 18, with a range of 0-91 surveys. Across participating facilities, 16 704 complete and valid surveys were obtained.

### Study sample

From 16 704 respondents, we excluded 12 respondents from very small facilities (with 5 or fewer beds) to focus on typical AL settings; we excluded 72 residents from facilities with a provisional license due to small numbers, which would lead to a lack of generalizability of pooled estimates to this subpopulation; and we excluded 1300 respondents with missing outcome (satisfaction) data. Our final analytic sample included 15 320 respondents (Supplementary Figure 1). Missingness across variables was minimal. Except for age (6.4% missing) and relationship with residents (12% missing), all other participants and facility characteristics had missing rates below 5%. Given the low proportion of missingness and the descriptive nature of the study, we conducted a complete case analysis.

### Measurement of family satisfaction

We evaluated 8 domains of family satisfaction: *Choice*, *environment*, *experience*, *food*, *housekeeping*, *needs*, *staff*, and *overall*. The domains have been validated and have good measures of reliability.^10^ Briefly, the *Choice* domain comprises 5 items about whether they have opportunities to provide input in their resident’s care, etc. The *Environment* domain comprises 3 items, including questions about whether the environment is safe. The *Experience* domain comprises items such as whether the family member feels welcome when they visit and whether they are comfortable voicing concerns. The *Food* domain comprises 3 questions around food variety and whether their resident enjoys mealtime/food. The *Housekeeping* domain comprises 4 items about whether the resident’s living space and overall facility are maintained and clean. The *Needs* domain comprises 7 questions about whether their resident is given opportunities to be independent, whether the family member receives timely updates, etc. The *Staff* domain comprises 7 items, including whether staff know and treat residents with respect, whether management responds to concerns, and staff knowledge of the care plan. The *Overall* domain comprises 3 items about grading the facility on an A-F scale, whether the family feels confident with resident care, and the likelihood of recommending this facility to another family. All FSS domain scores are measured on a 0-3 scale (0 = negative, 3 = positive). Because the original scale of 0-3 point scores may be less intuitive, we rescaled the domain-specific scores to a 0-100 scale for ease of interpretation.

### Ascertainment of survey respondent and facility characteristics

Participants’ characteristics in the survey included several respondent-reported demographic and relational factors, which we categorized as follows. *Age* was recorded as a 5-level categorical variable as follows: 18-54 years, 55-64 years, 65-74 years, 75 years or older, and missing. *Gender* was classified as male, female, or missing. Because the sample was predominantly White, *Race* was grouped as White, minoritized (including all racial and ethnic minorities), or missing (0.6% of the study sample). Additional predictors included family engagement characteristics. *Employment status* of the respondent was categorized as employed (full-time and part-time), retired, other (unemployed, student, homemaker), or missing. *Relationship to the resident* was classified as child, spouse, sibling, other relative (including friend, other relative, and guardian), or missing. *Visit frequency* was coded as once a week or more often, a couple of times a month, or no more than once a month, with missing data separately. *Communication frequency* was measured similarly, with response options including once a week or more often, a couple of times a month, no more than once a month, or not applicable if the resident was unable to communicate, with missing records separately. *Facility-level characteristics included* facility size, ownership type, dementia care designation, and geographic location. *Facility size* was categorized as small (6-25 residents), medium (26-100 residents), or large (more than 100 residents). *Ownership* was classified as for-profit or nonprofit/governmental/tribal. Facilities were further grouped based on whether they offered dementia care services. *Geographic location* was categorized as Twin Cities metro or rest of state.

### Statistical analysis

We summarized domain-specific and overall satisfaction scores, and characteristics of AL facilities and residents’ family members who responded to the FSS using descriptive statistics, reporting categorical variables as frequencies (percentages) and continuous variables as means with standard deviations. We used multivariable linear regression models to evaluate associations between residents’ family characteristics plus facility characteristics and the likelihood of having a valid quality of life score across the following domains: Experience, choice, needs, housekeeping, food, environment, staff, and overall. Model estimates were presented as regression coefficients (β) with 95% confidence intervals (CIs) and *p* values. To address the clustering of residents within facilities, we incorporated a random intercept for each facility in all models.^11^ To address the issue of multiple comparisons across domains and covariates, *p* values were adjusted using the Benjamini–Hochberg procedure as it controls the false discovery rate, which is preferable in this context where numerous correlated hypotheses are tested.^12^ This approach reduces the likelihood of false positives while retaining greater statistical power than more conservative corrections such as Bonferroni. All analyses were performed in R (version 4.3.3; R Foundation for Statistical Computing, Vienna, Austria) using RStudio (version 2024.02.29).

## Results

A total of 15 320 family members participated in the Minnesota Assisted Living Report Card FSS in 2024. As shown in Table 1, most respondents were in relation to residents from medium-sized (26-100 residents) facilities (56.0%), followed by large (100+) facilities (36.7%). More than half of the facilities were for-profit (61.2%), and three-quarters (75.4%) offered dementia care services. Respondents were evenly distributed between the Twin Cities metro (50.2%) and the rest of the state (49.8%). The largest age group of respondents was 55-64 years (35.9%), followed by 65-74 years (32.0%) and 18-54 years (15.2%). The sample was predominantly White (92.5%) and female (65.4%). Nearly half of the respondents were employed (43.3%) or retired (44.8%). Most respondents were the resident’s child (64.7%), with smaller proportions identifying as spouses (5.0%) or siblings (7.1%). Two-thirds (66.2%) reported visiting their family members at least once a week, and a similar proportion (65.5%) communicated with them weekly or more often, while 18.1% indicated that communication was not applicable because the resident was unable to communicate.

**Table 1:** Descriptive characteristics of the Family Satisfaction Survey participants: The Minnesota Assisted Living Report Card, 2024.

Variable	Response	**Overall (*N* = 15** **320)**
**Family Satisfaction Survey domain scores, mean (*SD*)**		
Experience		77.45 (17.5)
Choice		79.66 (17.17)
Needs		72.61 (20.37)
Housekeeping		80.08 (17.85)
Food		67.22 (24.1)
Environment		80.93 (17.6)
Staff		77.16 (18.63)
Overall		82.07 (21.2)
**Family respondent characteristics, *N* (%)**		
Age group (years)	18-54	2325 (15.2)
	55-64	5499 (35.9)
	65-74	4901 (32.0)
	75+	1613 (10.5)
	Missing	982 (6.4)
Race	White	14 175 (92.5)
	People of color	1054 (6.9)
	Missing	91 (0.6)
Gender	Male	4983 (32.5)
	Female	10 013 (65.4)
	Missing	324 (2.1)
Employment	Employed	6626 (43.3)
	Retired	6864 (44.8)
	Other	1106 (7.2)
	Missing	724 (4.7)
Relationship	Child	9906 (64.7)
	Spouse	773 (5.0)
	Sibling	1087 (7.1)
	Other	1723 (11.2)
	Missing	1831 (12.0)
Visit frequency	Once a week or more often	10 141 (66.2)
	A couple times a month	2380 (15.5)
	No more than once a month	2148 (14.0)
	Missing	651 (4.2)
Communication frequency	Once a week or more often	10 038 (65.5)
	A couple times a month	1059 (6.9)
	No more than once a month	2775 (18.1)
	Not applicable (resident is unable to communicate)	803 (5.2)
	Missing	645 (4.2)
**Facility characteristics, *N* (%)**		
Facility size	Small	1178 (7.7)
	Medium	8517 (55.6)
	Large	5625 (36.7)
Ownership	For-profit	9370 (61.2)
	Nonprofit	5950 (38.8)
Dementia care	Assisted living facility	3767 (24.6)
	Assisted living facility-dementia care unit	11 553 (75.4)
Geography	Twin Cities metropolitan area	7686 (50.2)
	Rest of state	7634 (49.8)

**Figure 1:**
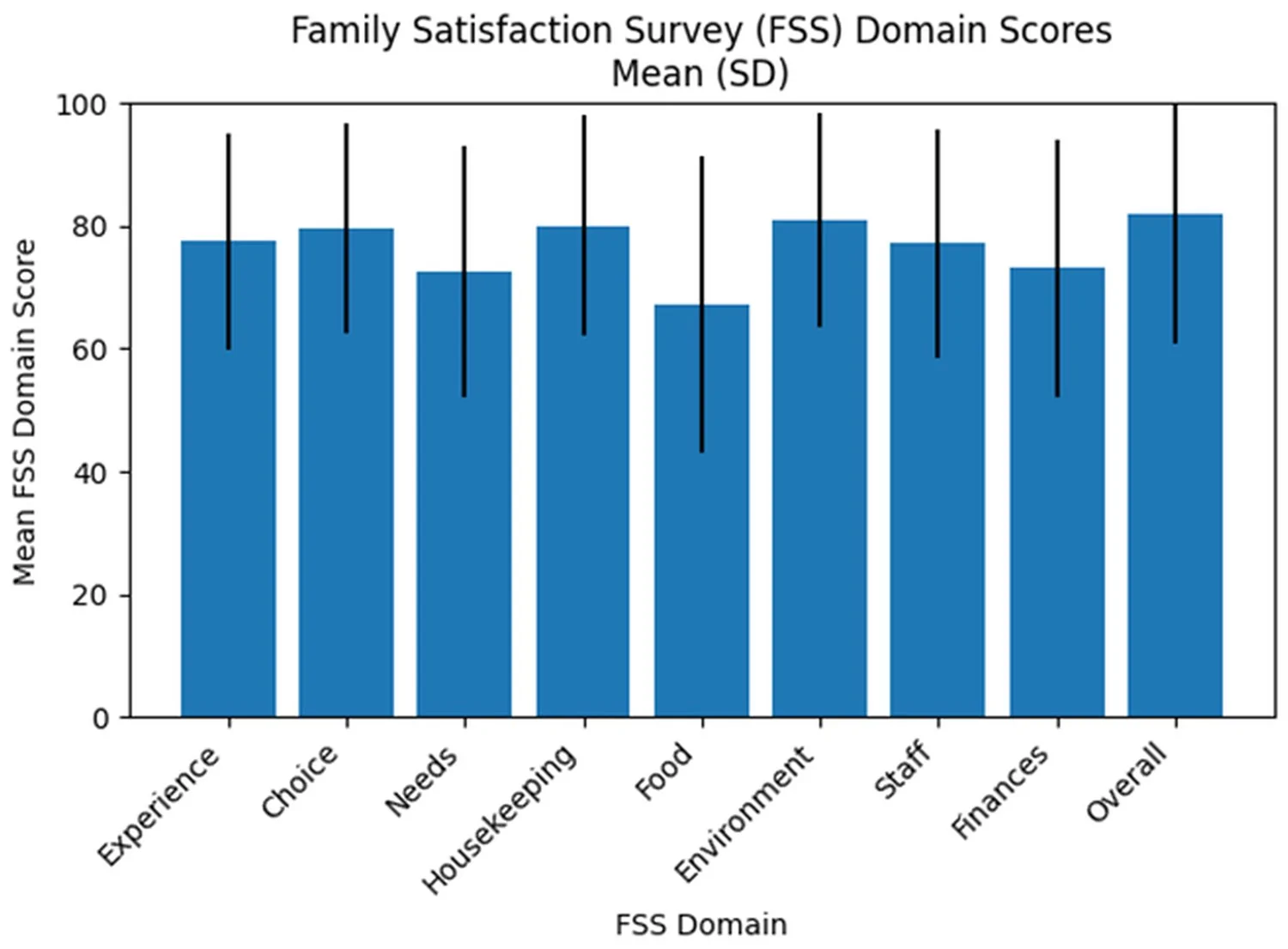
Domain-specific and overall family satisfaction scores: The Minnesota Assisted Living Report Card, 2024.

FSS scores (Table 1; Figure 1; Supplementary Figure 2) indicated generally positive perceptions across most domains, with mean ratings ranging from 67.22 to 82.07 on a 100-point scale. Among the 7 domains, *Overall* (mean = 82.07, *SD* = 21.2), *Environment* (mean = 80.93, *SD* = 17.6), and *Housekeeping* (mean = 80.08, *SD* = 17.85) received the highest average scores, suggesting favorable family impressions of the physical surroundings, cleanliness, and residents’ autonomy. The *Food* domain had the lowest mean score (67.22, *SD* = 24.1), indicating greater room for improvement in meal quality or dining experiences. Intermediate ratings were observed for *Experience* (77.45, *SD* = 17.5), *Staff* (77.16, *SD* = 18.63), and *Needs* (72.61, *SD* = 20.37).

At the family-member level (Table 2), respondents aged 18-54 and 55-64 reported significantly lower scores across all domains than those aged 65-74, whereas respondents aged 75 and older reported higher ratings across several domains. Compared to White residents, people of color consistently rated all domains lower than White respondents. Women also reported significantly lower scores than men across all domains. Compared with retired respondents, those who were employed reported lower satisfaction across multiple domains, including experience, choice, and staff; whereas those in other categories (eg, students, homemakers, and part-time workers) reported lower scores in experience and overall satisfaction. In terms of relationship to the resident, compared with children, respondents who were spouses and siblings reported significantly lower ratings across all domains except food. Families visiting less frequently (once a month or less) tended to rate experience and choice domains lower, although these associations were modest. Communication frequency also showed robust associations: compared with those communicating weekly, families communicating once a month or less reported significantly lower scores in all domains except food, but reported significantly higher scores for food.

**Table 2:** Family- and facility-level predictors of Family Satisfaction Survey domains from multivariable linear regression analyses: The Minnesota Assisted Living Report Card, 2024.

Predictor	Choice	Environment	Experience	Food	Housekeeping	Needs	Staff	Overall
**Facility size**								
Small	Ref.	Ref.	Ref.	Ref.	Ref.	Ref.	Ref.	Ref.
Medium	−2.427** (−3.905, −0.949)	−2.667*** (−4.139, −1.195)	−3.250*** (−4.828, −1.672)	−4.274*** (−6.181, −2.367)	−3.214*** (−4.698, −1.731)	−3.687*** (−5.469, −1.906)	−3.190*** (−4.824, −1.557)	−4.189*** (−6.019, −2.359)
Large	−2.053* (−3.757, −0.349)	−1.806* (−3.490, −0.121)	−2.965** (−4.809, −1.121)	−3.634*** (−5.774, −1.495)	−2.292** (−3.990, −0.595)	−3.539*** (−5.597, −1.481)	−2.822** (−4.713, −0.932)	−3.324** (−5.443, −1.206)
**Ownership**								
For-profit	Ref.	Ref.	Ref.	Ref.	Ref.	Ref.	Ref.	Ref.
Nonprofit	0.825 (−0.105, 1.754)	0.866 (−0.044, 1.776)	0.830 (−0.194, 1.853)	0.542 (−0.578, 1.661)	1.375** (0.458, 2.291)	0.975 (−0.151, 2.101)	0.720 (−0.317, 1.757)	1.694** (0.532, 2.857)
**Dementia care**								
ALF	Ref.	Ref.	Ref.	Ref.	Ref.	Ref.	Ref.	Ref.
ALF-DC	−1.041 (−2.120, 0.039)	−0.479 (−1.540, 0.582)	−1.023 (−2.203, 0.156)	0.422 (−0.901, 1.744)	0.037 (−1.031, 1.106)	−1.355* (−2.660, −0.049)	−1.195 (−2.396, 0.006)	−0.799 (−2.146, 0.547)
**Geography**								
TCM	Ref.	Ref.	Ref.	Ref.	Ref.	Ref.	Ref.	Ref.
ROS	−0.142*** (−0.171, −0.112)	−0.140*** (−0.169, −0.111)	−5.709*** (−6.797, −4.621)	−0.125*** (−0.160, −0.089)	−0.134*** (−0.163, −0.105)	−0.150*** (−0.186, −0.114)	−0.139*** (−0.172, −0.106)	−0.152*** (−0.189, −0.115)
**Age group (years)**								
65-74	Ref.	Ref.	Ref.	Ref.	Ref.	Ref.	Ref.	Ref.
18-54	−1.622** (−2.690, −0.555)	−1.511** (−2.608, −0.414)	−2.001*** (−3.066, −0.935)	−1.561* (−3.096, −0.026)	−2.034*** (−3.143, −0.926)	−2.383*** (−3.657, −1.108)	−1.855** (−3.014, −0.697)	−2.431*** (−3.728, −1.134)
55-64	−1.048** (−1.845, −0.252)	−1.081** (−1.900, −0.263)	−0.876* (−1.671, −0.081)	−1.364* (−2.509, −0.218)	−1.11 (−1.937, −0.283)	−1.656*** (−2.607, −0.706)	−1.116* (−1.980, −0.252)	−1.388** (−2.356, −0.421)
75+	0.901 (−0.274, 2.076)	0.963 (−0.245, 2.170)	1.445* (0.272, 2.617)	2.76** (1.071, 4.450)	2.103*** (0.883, 3.323)	1.591 (0.188, 2.994)	1.561 (0.286, 2.836)	1.896** (0.469, 3.324)
**Race**								
White	Ref.	Ref.	Ref.	Ref.	Ref.	Ref.	Ref.	Ref.
People of color	−3.481*** (−4.774, −2.188)	−2.731*** (−4.060, −1.402)	−3.746*** (−5.036, −2.455)	−3.568*** (−5.427, −1.709)	−3.745*** (−5.088, −2.403)	−4.413*** (−5.957, −2.869)	−4.232*** (−5.635, −2.829)	−5.724*** (−7.295, −4.153)
**Gender**								
Male	Ref.	Ref.	Ref.	Ref.	Ref.	Ref.	Ref.	Ref.
Female	−0.681 (−1.300, −0.063)	−0.805* (−1.441, −0.169)	−1.586*** (−2.203, −0.969)	−2.368*** (−3.257, −1.478)	−1.241*** (−1.883, −0.598)	−2.586*** (−3.324, −1.847)	−1.282*** (−1.953, −0.611)	−2.101*** (−2.853, −1.35)
**Employment**								
Retired	Ref.	Ref.	Ref.	Ref.	Ref.	Ref.	Ref.	Ref.
Employed	−1.119** (−1.878, −0.359)	0.126 (−0.654, 0.907)	−1.254** (−2.011, −0.496)	−0.209 (−1.301, 0.884)	0.382 (−0.407, 1.170)	−0.050 (−0.956, 0.857)	−1.064* (−1.888, −0.24)	−0.509 (−1.431, 0.413)
Other	−1.270 (−2.540, 0.001)	−0.060 (−1.366, 1.246)	−1.424* (−2.691, −0.156)	−1.589 (−3.417, 0.239)	−0.116 (−1.435, 1.204)	−0.667 (−2.184, 0.850)	−1.323 (−2.701, 0.056)	−2.307** (−3.850, −0.764)
**Relationship**								
Child	Ref.	Ref.	Ref.	Ref.	Ref.	Ref.	Ref.	Ref.
Spouse	−2.52** (−4.021, −1.019)	−2.222** (−3.764, −0.680)	−2.008** (−3.507, −0.509)	0.669 (−1.487, 2.825)	−1.645* (−3.203, −0.087)	−3.136*** (−4.928, −1.343)	−2.142** (−3.771, −0.513)	−3.335*** (−5.159, −1.511)
Sibling	−3.183*** (−4.330, −2.036)	−2.145*** (−3.323, −0.967)	−2.581*** (−3.727, −1.436)	1.356 (−0.291, 3.003)	−2.353*** (−3.543, −1.162)	−1.86** (−3.23, −0.491)	−1.855** (−3.1, −0.611)	−1.892** (−3.286, −0.498)
Other	−0.750 (−1.669, 0.168)	−0.828 (−1.772, 0.116)	−0.575 (−1.492, 0.343)	0.376 (−0.944, 1.696)	−0.137 (−1.091, 0.817)	−0.738 (−1.835, 0.359)	−0.595 (−1.592, 0.401)	−1.663** (−2.779, −0.547)
**Visit frequency**								
Once a week or more often	Ref.	Ref.	Ref.	Ref.	Ref.	Ref.	Ref.	Ref.
A couple times a month	−0.883* (−1.695, −0.071)	−0.302 (−1.137, 0.532)	−0.600 (−1.410, 0.210)	0.216 (−0.952, 1.384)	0.387 (−0.456, 1.230)	0.761 (−0.209, 1.73)	−0.03 (−0.911, 0.851)	1.147* (0.161, 2.134)
No more than once a month	−0.701 (−1.605, 0.203)	−0.35 (−1.279, 0.579)	0.131 (−0.771, 1.034)	0.133 (−1.166, 1.432)	0.521 (−0.417, 1.460)	0.839 (−0.241, 1.918)	0.035 (−0.946, 1.017)	1.534** (0.436, 2.633)
**Communication frequency**								
Once a week or more often	Ref.	Ref.	Ref.	Ref.	Ref.	Ref.	Ref.	Ref.
A couple of times a month	0.238 (−0.922, 1.398)	−0.025 (−1.218, 1.167)	0.305 (−0.853, 1.463)	3.214*** (1.545, 4.883)	−0.119 (−1.324, 1.086)	1.218 (−0.167, 2.603)	0.903 (−0.355, 2.162)	0.427 (−0.982, 1.837)
No more than once a month	−1.158** (−1.947, −0.368)	−2.836*** (−3.647, −2.025)	−1.114** (−1.902, −0.326)	5.991*** (4.857, 7.124)	−3.069*** (−3.888, −2.249)	−1.308** (−2.251, −0.366)	−1.077* (−1.934, −0.22)	-2.383*** (−3.343, −1.424)
Not applicable (resident is unable to communicate)	0.726 (−0.638, 2.089)	−0.638 (−2.038, 0.763)	0.311 (−1.049, 1.672)	6.673*** (4.713, 8.633)	−0.625 (−2.041, 0.790)	2.060* (0.433, 3.688)	0.621 (−0.858, 2.100)	1.675* (0.019, 3.331)

Domain scores range from 0 to100.

Abbreviations: ALF, assisted living facility; DC, dementia care unit; FSS, Family Satisfaction Survey; ROS, rest of state; TCM, Twin Cities metropolitan area.

*** *p* < .0001.

** *p* < .001.

* *p* < .05.

Facility-level characteristics showed several significant associations across family satisfaction domains (Table 2). Compared with small facilities, both medium and large facilities consistently demonstrated significantly lower scores across all domains, including experience, choice, needs, housekeeping, food, environment, staff, and overall satisfaction (*p* < .05). Nonprofit ownership was associated with higher ratings in housekeeping and overall satisfaction domains compared with for-profit facilities. No significant differences were observed by dementia care type. Geographically, facilities located outside of the Twin Cities metro areas had significantly lower scores than their counterparts across all domains, with the largest difference in the experience domain, indicating more favorable family-reported experiences at metropolitan facilities.

## Discussion

In this large, statewide sample of family members of AL residents, we found generally positive perceptions of care, with the highest satisfaction observed in overall facility grade, environment, and housekeeping domains, and the lowest satisfaction in the food quality domain. These findings align with prior research demonstrating that families value physical surroundings, cleanliness, and residents’ autonomy, while meals and dining experiences remain persistent areas for improvement in AL settings.^3–6^

Our results highlight the importance of family and resident characteristics in shaping perceptions of care.^3–6^^,^^13^ Younger family members and respondents of color consistently reported lower satisfaction across multiple domains, suggesting that expectations, cultural values, or prior experiences with healthcare and long-term care may influence evaluations.^3^^,^^6–8^^,^^13^^,^^14^ Female respondents also reported lower satisfaction than males, particularly regarding experience, needs, and overall perceptions, aligning with prior studies indicating gender differences in family caregiving roles and expectations.^13–15^ Similarly, the relationship to the resident mattered: spouses and siblings tended to rate nearly all domains lower than children, perhaps reflecting greater emotional proximity, caregiving involvement, and expectations for communication and responsiveness.^7^^,^^8^^,^^13–15^ Frequency of visits and communication showed nuanced associations, with less frequent visitation generally linked to lower experience and choice ratings, while limited communication due to resident impairment was associated with higher food and overall scores, highlighting the complex interplay between resident-family interaction and family’s satisfaction with different quality domains.^13–15^ Facilities can address these disparities by implementing structured, proactive family engagement strategies.^16^^,^^17^ These may include establishing regular care conferences, developing individualized communication plans based on family preferences, establishing standardized communication protocols including routine updates on resident status, use of digital communication platforms where appropriate, and designating consistent staff points of contact for highly involved family members.^16^^,^^17^ In addition, staff training focused on family engagement, expectation setting, and conflict resolution may help improve interactions with families who are more closely involved in day-to-day care.^16–18^

Facility-level characteristics were also strongly associated with family satisfaction, consistent with prior research.^3–8^ Medium and large facilities consistently received lower ratings across domains compared with small facilities. This pattern suggests that larger organizational size may introduce challenges related to care personalization, continuity, and relationship-building between staff and families. To mitigate these challenges, larger facilities may benefit from adopting organizational models that promote smaller, more homelike care environments, such as unit-based or household models within facilities.^19^ Consistent staff assignment, reduced staff turnover, and workflow redesign to support more frequent and meaningful staff—family interactions may further enhance relationship continuity and trust, which are central to family satisfaction in residential care.^20^ Nonprofit ownership was associated with higher satisfaction in housekeeping and overall domains, consistent with prior studies linking nonprofit status to higher perceived quality in residential long-term care.^21–23^ Interestingly, in our analyses, dementia care availability was not associated with differences in satisfaction. A potential explanation for this may be that families prioritize day-to-day experiences and interactions over specialized care labels, because ongoing interpersonal contact, communication, and routine care interactions constitute core aspects family members value in quality assessments.^6^^,^^24^^,^^25^ Geographically, facilities in the Twin Cities metropolitan areas had higher ratings than those in the rest of the state (comprising mostly non-metropolitan areas), likely reflecting differences in staffing, resources, or access to services. These findings contrast with previous research, which reports higher quality in rural AL facilities compared with urban AL facilities.^26^

These findings have important implications for practice, policy, and research. For practice, our results highlight key areas for targeted quality improvement. Efforts to enhance food quality, resident experience, and communication may yield meaningful gains in family satisfaction.^3–6^ Smaller facilities and nonprofit organizations appear to foster higher family-reported satisfaction, suggesting that elements such as homelike environments, staffing patterns, and organizational culture warrant attention in larger or for-profit settings.^3–6^ Facilities in non-metropolitan areas may require additional support to achieve comparable satisfaction levels.^21^^,^^23^^,^^27^ Providers should also consider tailored strategies to engage younger family members, families of people of color, and spouses or siblings, recognizing that these groups may have different expectations or priorities.^3^^,^^7^^,^^8^^,^^13–15^^,^^27^ For policy, the findings support the value of publicly available family satisfaction measures, such as the Minnesota Assisted Living Report Card, in monitoring and incentivizing quality improvements across facilities of diverse sizes, ownership types, and geographic contexts.^9^ Policymakers may consider integrating family satisfaction metrics alongside traditional quality indicators to guide regulatory oversight, resource allocation, and targeted interventions. For research, these results highlight the need for longitudinal studies to examine causal pathways between facility characteristics, family engagement, and satisfaction outcomes.^3^^,^^6^ Further work is also warranted to explore the experiences of racially and ethnically diverse families, residents with cognitive or functional limitations, and those in states with differing regulatory frameworks, to ensure generalizability and equity in AL quality improvement efforts. Future surveys should consider including questions to capture language preferences and potential language barriers, which may influence family satisfaction and communication with staff. Including such items would improve the ability to identify disparities in care experiences, particularly in more linguistically diverse settings, and inform targeted interventions to enhance equitable, culturally and linguistically appropriate care in AL facilities.^13^^,^^21^^,^^24^

### Strengths and limitations

Strengths of this study include its large, population-based sample and the integration of individual- and facility-level data, allowing a nuanced, multilevel understanding of factors influencing family satisfaction. Our study has several limitations. First, the cross-sectional survey design precludes causal inference. Second, although the survey achieved broad statewide coverage, respondents may differ from non-respondents in unmeasured ways, introducing potential selection bias. Third, while we adjusted for multiple family- and facility-level covariates, residual confounding from unmeasured factors such as resident cognitive status, functional limitations, staffing ratios, or intensity of family involvement remains possible. Future research should consider more advanced analytic approaches, such as longitudinal designs, causal inference methods, and multilevel or mediation analyses, to better disentangle the pathways through which individual, relational, and facility factors concurrently influence family satisfaction. Fourth, data were drawn exclusively from Minnesota, which, despite its comprehensive AL oversight and the statewide Report Card system, may limit generalizability to states with differing AL regulations or reporting frameworks. However, as noted, currently Minnesota is the only state with such unique data. Despite these limitations, this study represents one of the largest and most comprehensive examinations of family- and facility-level correlates of satisfaction in AL, offering actionable insights to inform targeted quality improvement and family-centered care strategies.^3^^,^^6^

## Conclusions

Family- and facility-level characteristics, including respondent age, race, gender, relationship to the resident, frequency of visits and communication, facility size, ownership type, and geographic location, were significantly associated with family satisfaction across multiple domains. Family satisfaction in AL is shaped by a complex interplay of individual, relational, and facility factors. These associations should be confirmed in future longitudinal studies with causal inference methods to inform the development and evaluation of targeted interventions and policy initiatives to assure equitable, person-centered care in AL facilities. Understanding and acting on these is critically important at this juncture, given the growing importance of AL in the suite of long-term care options.

## Supplementary Material

igag067_Supplementary_Data

## Data Availability

The data used in this study can be acquired from the Minnesota Department of Human Services upon reasonable request and following their data use agreement. The study was not pre-registered.
