# Supplementary material for: Examining family and facility characteristics associated with family satisfaction in assisted living: the Minnesota Assisted Living Report Card project
Source: Innov Aging. 2026 Jun 23;10(9):igag067. doi: 10.1093/geroni/igag067 (PMC13459960; doi:10.1093/geroni/igag067)

Innovation in Aging **Supplementary Material: Shippee et al. Examining family and facility characteristics associated with family satisfaction in assisted living: The Minnesota Assisted Living a Card project.**

Supplementary Figure 1. Participant selection flow for the Family Satisfaction Survey: The Minnesota Assisted Living Report Card project.

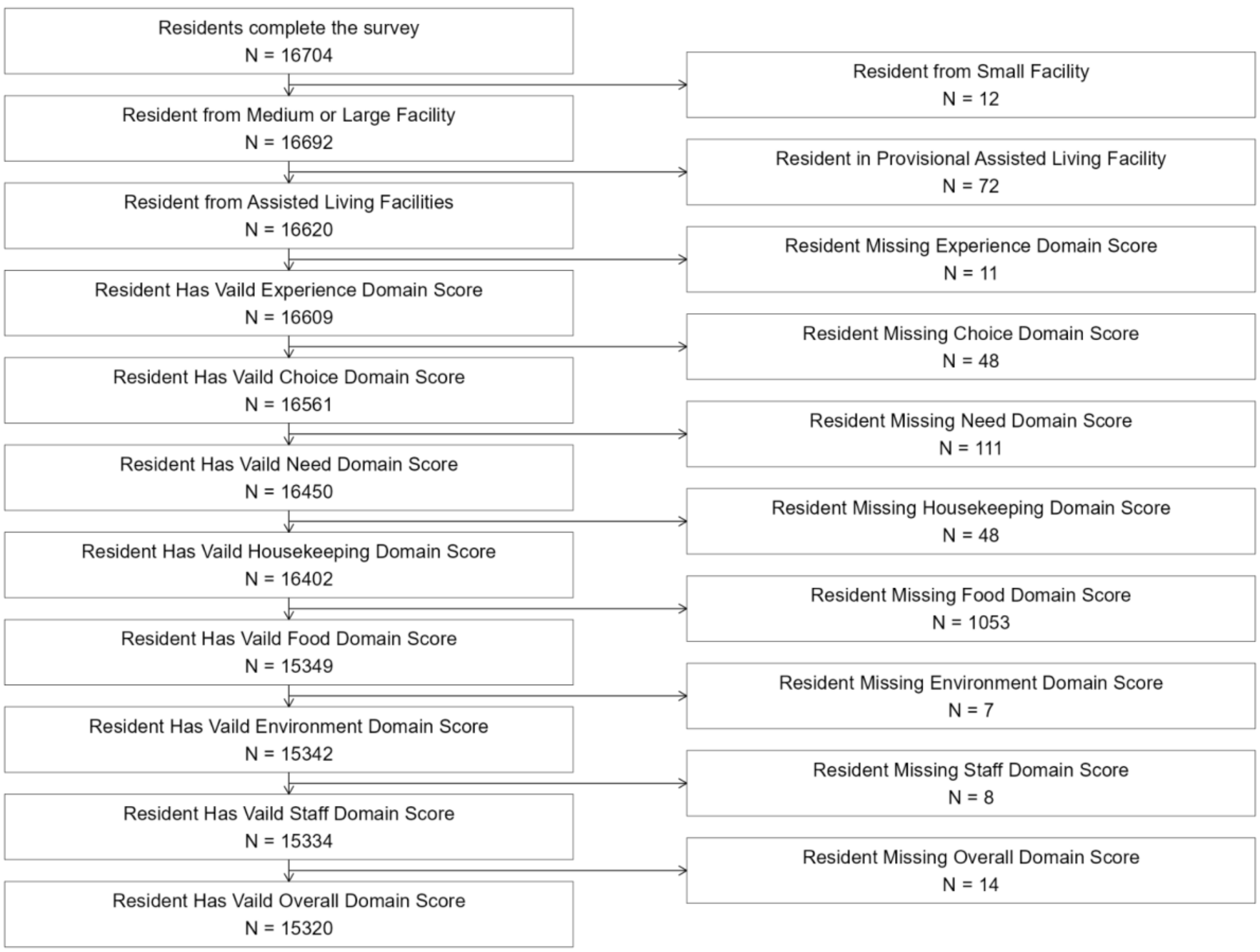

Supplementary Figure 2. Histogram showing the spread of scores for each of the seven domains of the Family Satisfaction Survey: The Minnesota Assisted Living Report Card project.

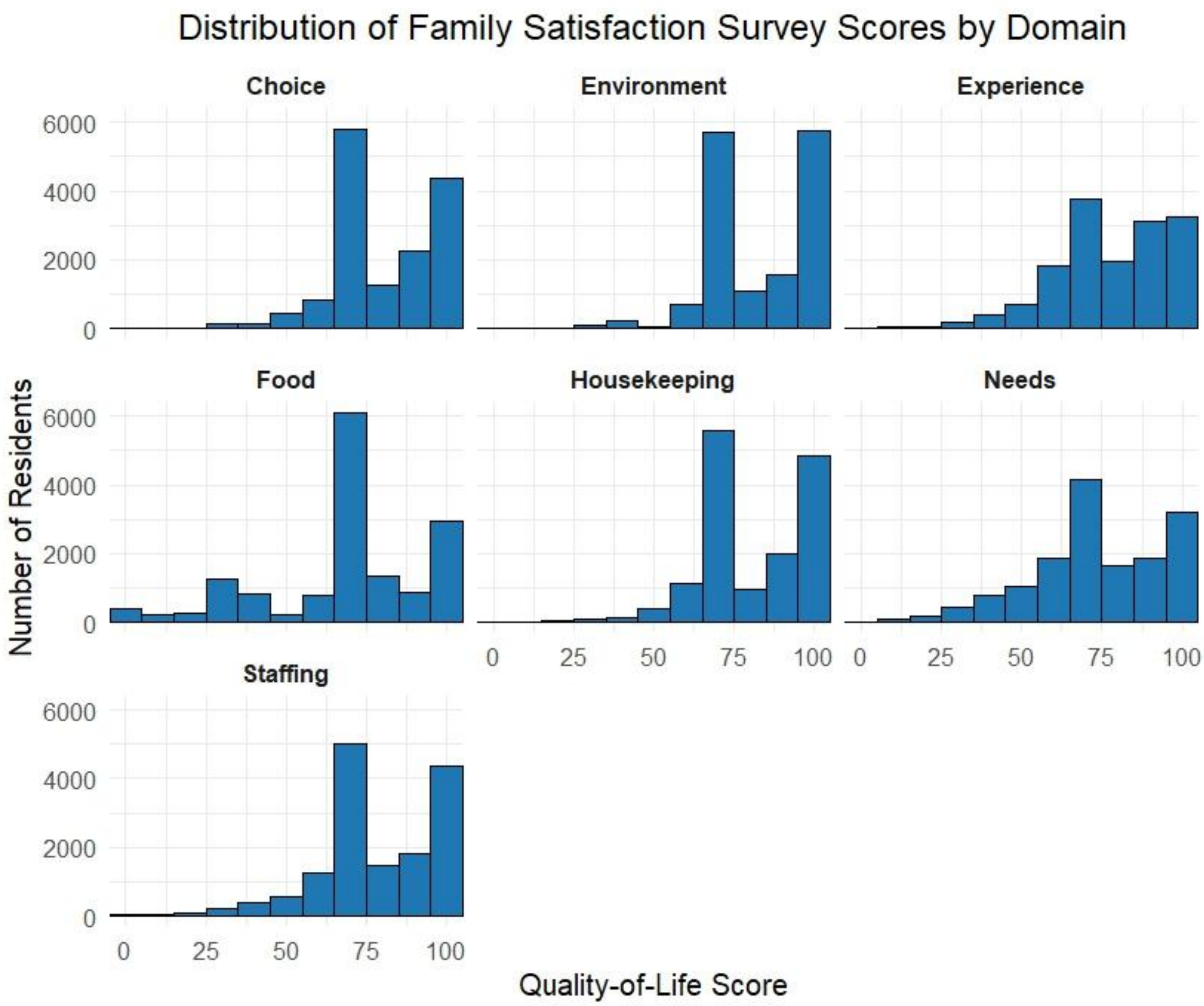

Supplement: Supplementary file 1 [file igag067_supplementary_data.pdf]
